# Forensic investigative issues in a fireworks production factory explosion

**DOI:** 10.1007/s00414-021-02564-5

**Published:** 2021-05-12

**Authors:** Gennaro Baldino, Chiara Stassi, Cristina Mondello, Antonio Bottari, Stefano Vanin, Elvira Ventura Spagnolo

**Affiliations:** 1 Section of Legal Medicine, Department of Health Promotion Sciences, Maternal and Infant Care, Internal Medicine and Medical Specialties (PROMISE), University of Palermo, Via del Vespro, 129 90127 Palermo, Italy; 2 Section of Legal Medicine, Department of Biomedical and Dental Sciences and Morphofunctional Imaging, University of Messina, Via Consolare Valeria, 98125 Gazzi, Messina Italy; 3 Section of Radiological Sciences, Department of Biomedical and Dental Sciences and Morphofunctional Imaging, University of Messina, Via Consolare Valeria, 98125 Gazzi, Messina Italy; 4 DISTAV, University of Genova, Genova, Italy

**Keywords:** Fireworks-related death, Forensic, Explosions, Blast injuries

## Abstract

Since their discovery in ancient China, fireworks rapidly spread throughout the world, where they have always been used to celebrate either popular or private events. Their use is nonetheless related to several risks, especially within production factories, since several injuries or even death can occur following an accidental ignition. In cases of major disasters related to fireworks explosions, stating the accidental or intentional nature of the event might prove challenging, thus raising the need of a multidisciplinary approach. In this regard, we here discuss the case of an accidental explosion that occurred in a fireworks production factory, accountable for five deaths and two hospitalisations.

## Introduction

Fireworks are explosive items containing a mixture of chemicals—mainly potassium nitrate, pulverised charcoal and sulphur—whose ignition generates spectacular and colourful light and sound effects. Casually born in China while managing several substances in an attempt to obtain the elixir of long life, fireworks soon spread throughout the world where they were used to celebrate either private, popular, cultural or religious events [1–7].

Their use is not, however, risk-free: When fireworks are improperly managed, the following explosion can cause severe injuries or, not infrequently, lead to death. For this reason, several national and European laws and guidelines have been issued over the years, in order to regulate both the sale and the handling of pyrotechnic artifices [4, 5, 8–11].

For the variability of the lesions found, explosion-related injuries are usually referred to as “compound injuries”: The blast wave effect on the body is responsible for contusions, lacerations, fractures, amputations, multi-organ damages; other injuries, resulting from direct and/or indirect mechanisms, include burns due to high temperatures, inhalation injury due to the toxic and hot gases released and injuries ascribed to the collapse on the body of the structures where the explosion occurs [2–4, 8, 9, 12–15].

According to literature, explosion-related deaths are not uncommon events, and are most frequently associated with terrorist or military activities; in this context, fireworks-related deaths account for just a few of all cases, being usually ascribed to suicidal attempts or accidents related to their production or use for fun and entertainment [4, 9, 14–19].

We here report the case of an accidental explosion of a fireworks production factory involving seven people, five of which died while the other two were hospitalised for serious wounds and internal injuries. Since from a preliminary judicial investigation a doubt arose on the accidental or intentional nature of the event due to recent contrasts between an employee hired without a regular contract and the factory’s owner, a multidisciplinary expertise has been requested in order to (1) evaluate the compatibility between the explosive event and the lesions observed on the victims and (2) shed light on the event’s dynamic.

## Case description

**Fig. 1:**
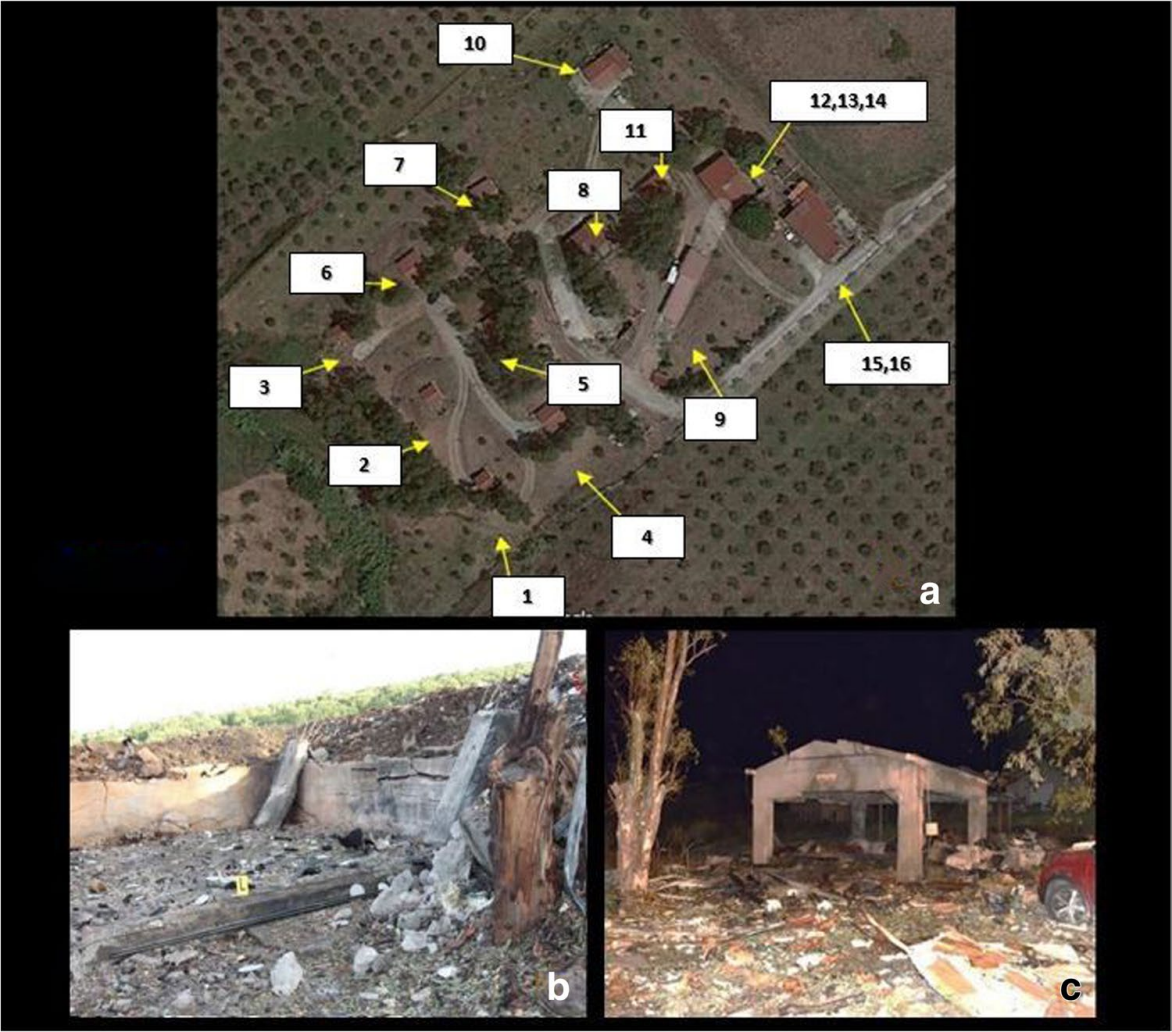
Satellite overview of the buildings of the fireworks factory (**a**), with a detail of buildings no. 7 (**b**) and 8 (**c**), destroyed by both the explosion and fire

**Fig. 2:**
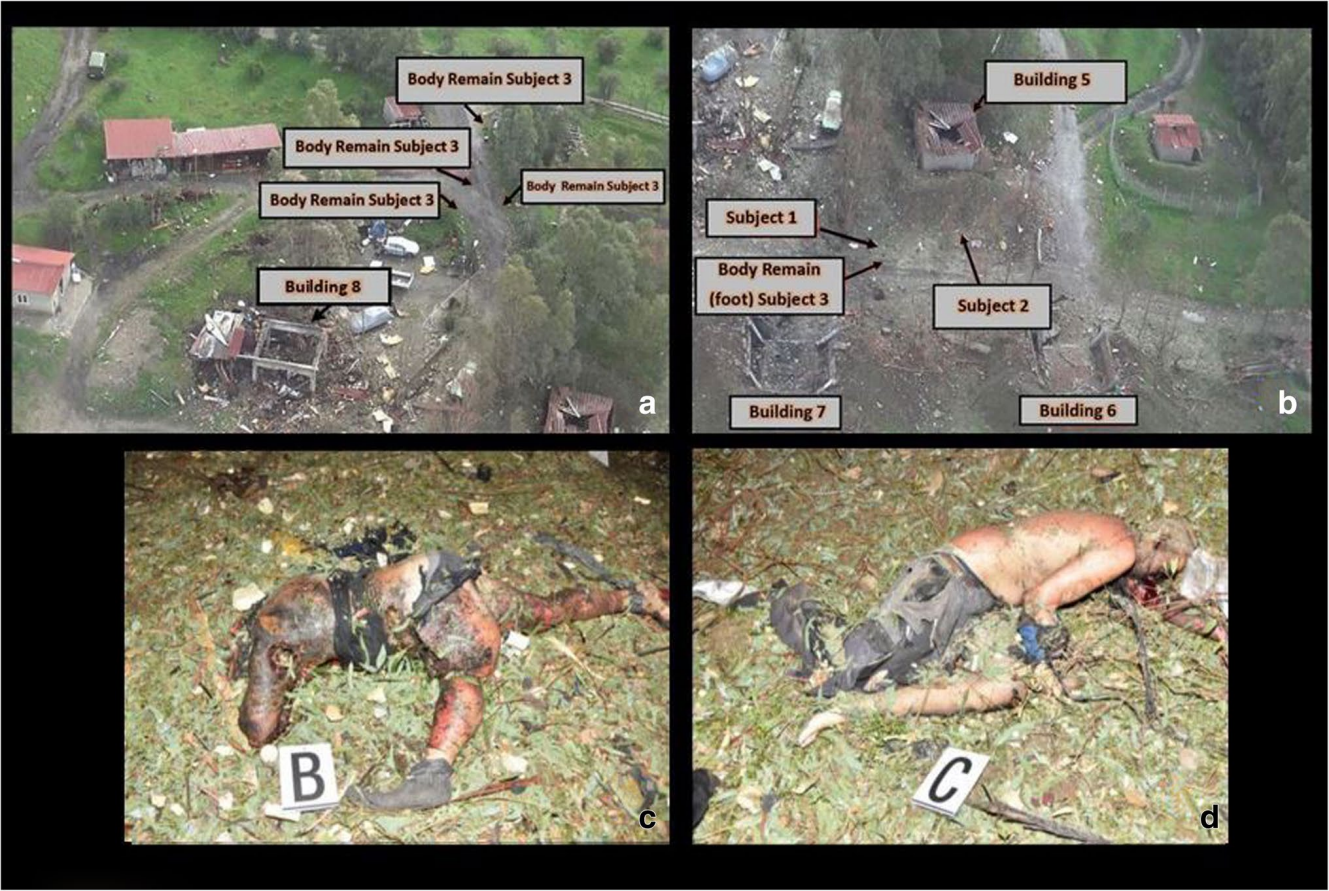
**a** and **b** Detail of the buildings involved in the explosion, two of which (buildings no. 6 and 7) completely destroyed. The cadavers of *subjects 1* and *2* were recovered, respectively, 12 m and 23 m from building no. 7; in between the two cadavers, a foot of *subject 3* was also recovered. Other body remains of *subject 3* were found within 30–40 m from building no. 8. **c** and **d** The bodies of *subjects 1* and *2* as they were found at the site of the accident

In November 2019, seven people were involved in a violent explosion that occurred within a fireworks factory: five of them were workers engaged in the installation of sliding gates to the factory buildings according to the latest safety regulations; the other two were administrative employees. The factory consisted of a total of 16 buildings: Buildings number 6 and 7 were completely destroyed by the explosion, and building number 8 also caught fire (Figs. 1 and 2); all the other buildings were affected by minor damages due to the deflagration-related blast wave—whose extent was proportional to the distance from the epicentre. Three workers and an employee died immediately: The corpses of two of the three workers—*subjects 1* and *2*—were found nearby the buildings number 6 and 7; several body parts of the third worker (*subject 3*) were spread not only in the area surrounding the same buildings, but also in the surroundings of building number 8 and beyond (Figs. 1a and 2); the employee’s corpse—*subject 4*—was found quite completely charred in proximity of building number 8 (Table 1). A fourth worker—*subject 5*—died while transported in severe conditions to the nearest hospital, while the second employee and the last worker—*subjects 6* and *7*—were transported to the hospital reporting major burns, minor fractures and other minor lesions and, once undergone adequate care, were discharged.

**Table 1:** Main circumstantial information, laboratory and toxicological findings in the five dead victims to evaluate the cause of death

Subject	Sex	Age	Position at the disaster site	External examination	DNA profile matrix	HbCO%	Cause of death
1	M	36	Nearby buildings no. 6 and 7	Smashed scalp; burns, bruises and abrasions all over the body surface	-	8.2%	Explosion-related
2	M	23	Nearby buildings no. 6 and 7	Head, neck and abdomen lacerations; burns, bruises and charred areas on the limbs	-	5.3%	Explosion-related
3	M (confirmed by DNA profiling)	34	Remains spread in proximity of buildings no. 6, 7 and 8	Dismembered body	Bone and muscle remains	-	Explosion-related
4	F	71	Nearby building no. 8	Almost completely charred; burns and lacerations of the head	-	8.2%	Explosion-related; charring
5	M	39	Dead while transported to the hospital	Burns and lacerations all over the body; exposed right tibial fracture	-	4.3%	Explosion-related

**Fig. 3:**
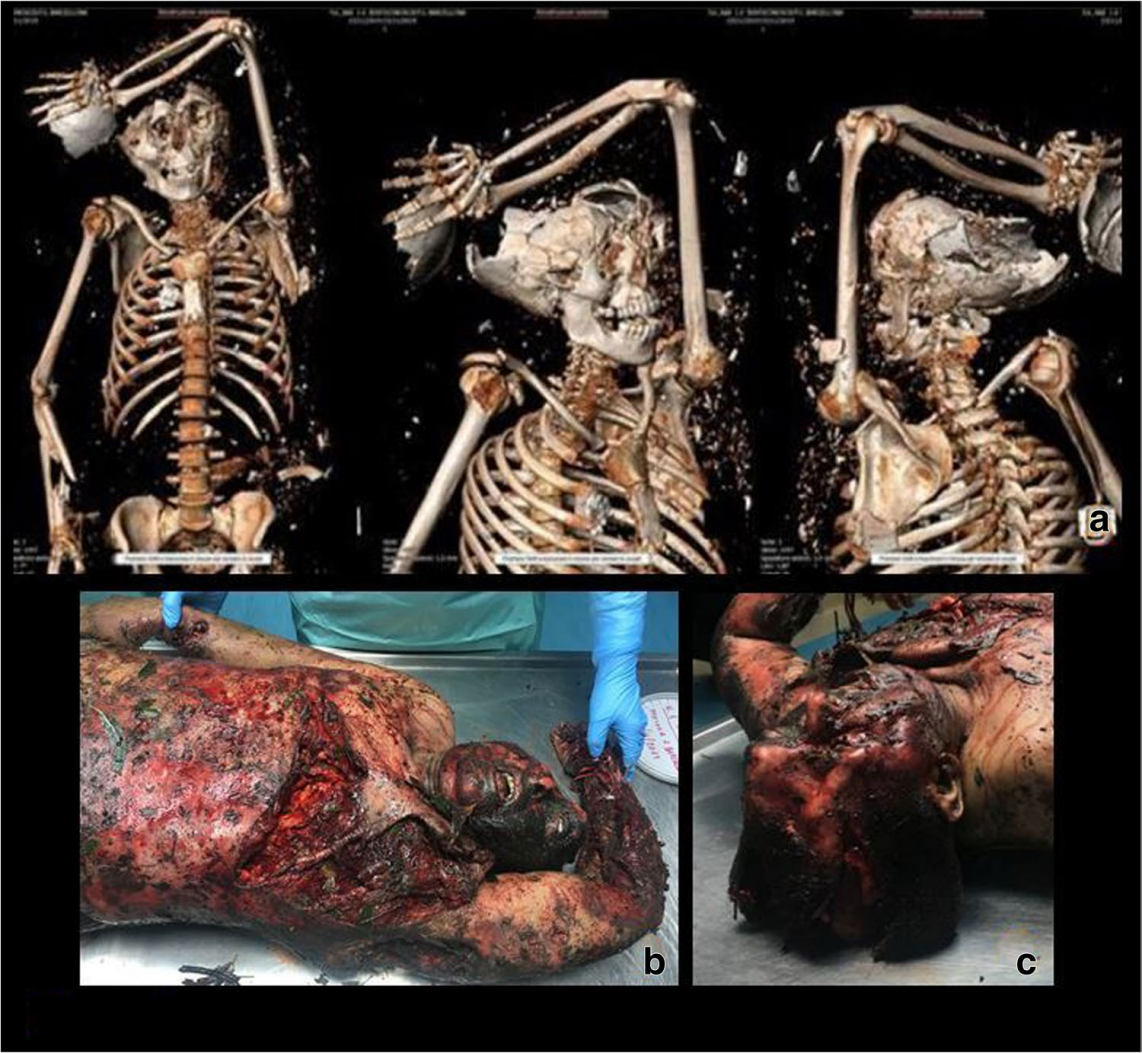
Subject no. 1: 3D CT scan (**a**) and details of the lesions detected on external inspection (**b** and **c**)

**Fig. 4:**
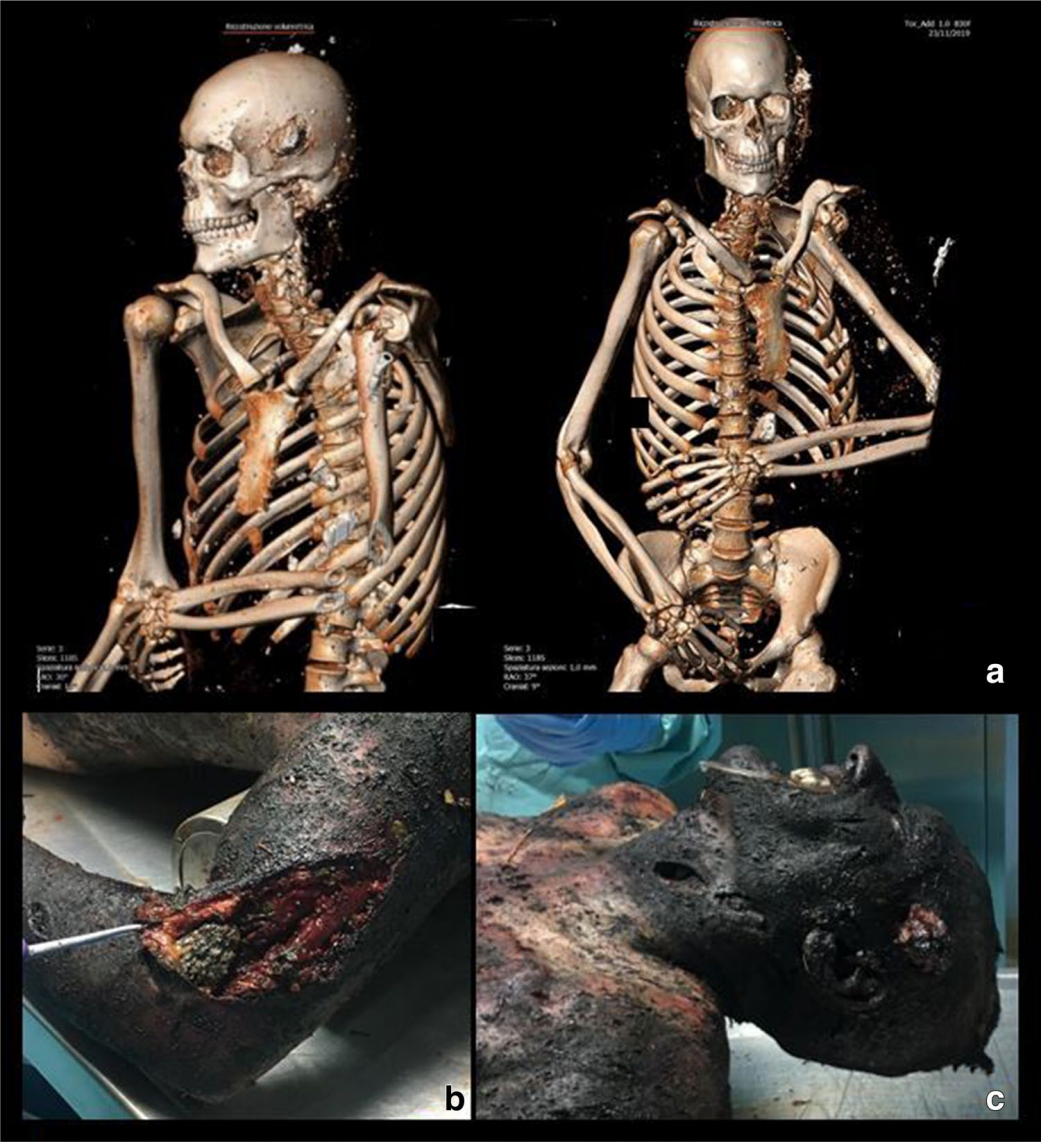
Subject no. 2: 3D CT scan (**a**) and details of the lesions detected on external inspection (**b** and **c**)

Prior to autopsies, 3D CT scans were performed in *subjects 1*, *2*, *4* and *5* both for a better understanding of the internal lesions and to detect the eventual presence of retained foreign bodies and/or unexploded ordnances (Figs. 3a and 4a). Widespread fractures were present in each case, and wide lacerations of the abdominal wall were observed in *subject 2*, while the presence of several foreign bodies was detected in *subjects 1*, *2* and *4*. Specifically, concrete foreign bodies released by the exploded buildings were detected in the right ribcage of *subject 1* (Fig. 3a), in the left temporo-parietal bone, in the anterior abdominal wall and in the subcutaneous planes of almost all districts of *subject 2* (Fig. 4a); a plastic foreign body was detected in the left parietal bone of *subject 4* (Table 2).

**Table 2:** Main 3D CT, external inspection, main cadaveric inspection and histological findings

	Subject 1	Subject 2	Subject 3	Subject 4	Subject 5
3D CT	- Cr and Mf – Frac - Rc Frac - FB in the Rc - UL – Frac - LL – Frac - Vc – Frac (D4)	- Cr Frac, FB retained in the cerebral parenchyma - Rc and LL Frac - Lac and FB in the AR - Small FBs in all districts	-	- Cr and Mf Frac - Rc Frac - Vc Frac (L2-L4-L5) - LL Frac	- Cr Frac - Rc Frac - UL Frac - LL Frac
External inspection	- BSD - DB and Ab - Smashed scalp with brain residues split outside - Extruded right eye bulb - Lost anatomical facial profile	- BSD - Cr Frac and FB; - H in the temporo-parietal areas - Lac of the left latero-cervical region - Lac in the AR - B and Ab in LL - B in UL - Ch LL	- Cr Frag - Mandibular bone with 2 dental elements - Mf Frag - Rc Frag - Vc Frag - UL Frag - LL Frag - Small Frag of muscular tissue - P parenchyma Frag	- Diffuse Ch; - Fighter attitude; - Burnt hair; - Mf Dis; - Cr Lac and H; - Small plastic object, roughly round-shaped, moulded within the parietal bone	- BSD; - LoS helix and anti-helix; - Mf B and Dis; - B throughout the body; - Cr Lac and B; - Mf, UL and LL Ab and grazes; - Lac and Frac LL
Cadaveric inspection	- Rupture of the right ventricle anterior wall - Multiple lac of lungs, liver and spleen	- Multiple subcutaneous and muscle HI - Sub-pleural E - Rc HI - Sub-epicardial HI - Residues of yellowish, pulpy, material in the oesophagus	-	- Cr HI - LoS left parietal hemisphere - Rotated mandibular arch - Rc HI - Pleural adhesions - Vy	- Black-smoky dirt was found in the oral cavity and airways - Cr HI - Pleural effusion - P parenchymal thickening
Histological findings	- Cerebral Oe - Er of glottis and trachea tunica propria and submucosa - P Co and Oe - P H and PE - Right ventricle subepicardial H - Cardiac Mif with focal contracture bands - Nec of the proximal renal cortical convolutes	- Diffuse subarachnoid H - Neuronal Oe - P S and Oe - Focal DA - MH - Splenic Hyp - Acute tubular Nec of renal cortex	- Hyperkeratotic seborrheic keratosis upon removal of the foot skin - H Oe and DA, with siderocytes in its context	- Subarachnoid H - Neuronal Oe - Brain stem acute S - MH associated with replacement, perivascular fibrosis and fibromuscular dysplasia of the small intramural coronary vessels - Slight Er of glottis and trachea tunica propria and submucosa - PE - Steatosis - Tubular Nec of the renal cortex	- Acute brain S and brain stem H - MH and Mif - P—H and Oe with DA - FE - Muciparous metaplasia of the bronchial epithelium - Spinal micro-embolism - Acute liver S with small outbreaks of turbid pathosis - Acute tubular Nec of the renal cortex and acute medullary S

Ab = Abrasions; AR = Abdominal region; B = Burns; BSD= Body surface dirty, with debris, combustion particles and foliage; Co = Congestion; Ch = Charring; Cr = Cranial; DA = Desquamative alveolitis; DB = Diffuse burns; Dis = De-epithelialization; E= Ecchymoses; Er = Erosion; FB = Foreign bodies; FE= Focal emphysema; Frac = Fractures; Frag = Fragments; H= Haemorrhage; Hyp = Hyperplasia; I= Infiltration; HI = Haemorragic infiltration; Lac = Lacerations; LL= lower limbs; LoS = loss of substance; Mif = Myofibrillolysis; Mf = Maxillo-facial; MH = Myocytes hypertrophy; Nec = Necrosis; Oe = Oedema; P = Pulmunary; PE= Pan-lobular emphysema; Rc = Rib cage; S = Stasis; UL = Upper limbs; Vc = Vertebral column; Vy = Ventricular concentric hypertrophy

Autopsies were performed 24 h after death in *subjects 1*, *2*, *3* and *4*, and 48 h after death in *subject 5*, who died while transported to the hospital; forensic investigations were completed by histopathology and toxicological assays. Upon external inspection, all the victims’ bodies and remains appeared dirty, with debris, combustion particles and foliage. Widespread lacerations, abrasions, burns from II to III degree and charred areas were detected on all subjects (Figs. 2c and d, 3b and c, 4b and c); as for *subject 4*, it was found almost completely charred, in a fighter attitude, with burnt hair and large areas of de-epithelialization on the face. *Subjects 1* and *3* presented cranial smash. A broken, exposed, right tibial fracture was also detected on *subject 4*. A detailed list of the autopsy and histological findings is provided in Table 2.

On both cadaveric inspection and histopathology, unspecific signs were observed, mainly consisting on haemorrhagic infiltrations and congestion of several organs; multiple lacerations of the lungs, liver and spleen were detected in *subject 1*, while just few bone and tissue remains were detected and collected in *subject 3*. Being the body of *subject 3* completely dismembered, and thus unrecognisable, subsequent genetic investigations were carried out on its remains, making it possible to trace the identity of the victim, which matched that of one of the five workers. The toxicological analyses on peripheral blood samples belonged from subjects 1, 2, 4 and 5 were performed revealing low levels of carboxy-haemoglobin (< 10%). All specimens resulted negative for alcohol and drugs.

## Discussion

Fireworks are a type of explosives which act by generating, upon ignition, a compression of the surrounding air, whose particles accelerate and heat, thus provoking an increase in the atmospheric pressure and temperature—the so-called blast wave—responsible for severe injuries [9, 16].

A possible explanation of the increasing trend of fireworks-related injuries lies in an easier accessibility due to the commercialization of the so-called class C fireworks, which are usually thought to be safe [8]. Very few cases are described in literature reporting their use for suicidal attempts, mainly by insertion inside the oral cavity, with subsequent death and disfigurement of the craniofacial structures [14, 17, 20]. Much more frequent are fireworks-related accidents, which recognise as main causes improper use by untrained people, handling in absence of adequate safety precautions within production factories or management-independent accidents. When the energy released by the explosion is high, the blast wave effects can be devastating both in terms of morbidity and mortality. This situation is more likely to occur in a working context, most frequently within confined spaces [1, 2, 5, 6, 14, 16, 18, 19].

On the whole, explosion-related injuries can be classified into four categories: Primary injuries are those related to the blast wave effect, leading to a major damage of gas-filled organs and air-fluid interfaces (e.g. lungs, gastrointestinal tract, internal ear); secondary injuries are those related to the penetrative effect of primary and secondary fragments released after the explosion; tertiary injuries are related both to the impact of the body, when displaced by the blast wave, towards surrounding structures, and/or to the collapse of the structure on the body (e.g. blunt injuries, concussions, crush syndromes); quaternary injuries are related to indirect damage mechanisms, including toxic gas inhalation, burns and environmental contamination [9, 12, 16, 21].

The *post mortem*, histological and toxicological investigations carried out on the five dead workers allowed us to detect all four classes of explosion-related injuries. Lung injuries considered blast-related included acute haemorrhagic oedema (*subjects 1*, *3* and *5*), pan-lobular (*subjects 1* and *4*) and focal (*subject 5*) acute emphysema and acute broncho-acinar haemorrhage in *subject 4* (Table 2). A bilateral tympanic perforation, another blast-related injury, was detected in *subject 6*, one of the two survivors. Some of our findings are also in agreement with Romolo et al. statements [12] according to which, although the homogeneous density of solid organs usually protects them from the action of the blast wave, when the blast load is high and the explosion is very close to the body, solid organs can suffer injuries as well (e.g. lacerations, ruptures). In this case, liver and spleen lacerations were detected in *subject 1*, while every organ of the body was destroyed in *subject 3*, of which only a few body remains were found. In each case, the inhalation injury was excluded given the low levels of carboxy-haemoglobin found in the victims’ blood samples (reference values < 10%) and the absence of soot in the airways, except for *subject 5*—the one who died while transported to the hospital—where just few traces of soot were found in the oral cavity and upper airways [22]. No alcohol or drugs were detected in any blood sample.

*Subject 3* is a typical example of the fact that, under the effect of the explosive phenomenon, human bodies can get completely dismembered, thus raising a critical problem: the correct identification of the subject. In such a context, a combined application of different techniques (DNA fingerprinting, comparison of dental structures) becomes of utmost importance [23, 24]. While the identification of four of the five victims was relatively easy due to the recognition of maintained physical features and/or worn objects (necklaces, bracelets, etc.), the identification of *subject 3* revealed challenging, since he was totally dismembered: The subsequent DNA profiling of the remains, once collected, allowed their attribution to a same individual, while the comparison to the DNA profiles of both parents made it possible to match the identity of the victim with that of one of the men working at the factory, a 34-year subject.

An equally important issue in cases of major explosions relates to the differentiation between an accidental and an intentional event. Even if, based on preliminary investigations, in the present case any element suggested that the explosion could be intentionally caused by third parties, a fire investigative unit survey was requested in order to elucidate the dynamics of the explosion and evaluate the presence of a compatibility with the circumstantial data provided by the judicial authority, the positions of the bodies at the site of discovery and the lesions found.

According to the report produced by the engineers of the fire investigative unit, a first explosion occurred at building number 7—used as deposit for fireworks dyes—where four workers were engaged in activities aimed at the installation of a sliding gate. During the survey on the remains of the building, which was otherwise destroyed, an extension cable still connected to the power cubicle was found departing from the ejected superior beam. On the same beam, several squared iron supports—used to weld the metal guide where the gate would slide—were found applied by means of a chemical anchor; welding signs were detected on one of the iron supports, thus confirming the ongoing gate installation. Given the absence of electricity in building number 7, the electric cause was excluded. Instead, it has been postulated that the deflagration would be consequence of the production of welding sparks in an area with combustible-oxidising atmosphere; the ignited atmosphere would thus act as a fuse for a domino effect which involved several buildings of the factory: The mainly affected were buildings number 6 and 8 (used, respectively, as fireworks deposit and fireworks production station) which, being very close to building number 7, were completely destroyed as well; Specifically, building number 6 exploded, while building number 8 also caught fire.

Such a reconstruction is in accordance with the forensic surveys: The cadavers of *subjects 1* and *2*—who were referred to be working nearby buildings number 6 and 7—and the remains of *subject 3*—who was referred to be engaged in the welding activity at building number 7—were found in the surroundings of buildings number 6 and 7; *subject 5*, who died while transported to the hospital, was referred to be working for the gate installation nearby buildings number 6 and 7 as well; at last, the discovery of the charred cadaver of *subject 4* close to building number 8 (where she was referred heading towards as the explosion occurred) is in accordance with the fact that the structure caught fire. In light of the present reconstruction, confirmed the absence of any element suggesting that the explosion could be intentional, and excluded causes of death different from an explosion-related one, the accidental nature of the event was thus validated.

As well as in the present case, particular contexts exist in which the forensic investigations show some limits; for this reason, the achievement of a correct and precise reconstruction of the dynamics of certain events cannot be achieved without a multidisciplinary approach in which different professional profiles, as well as a thorough analysis of the circumstantial data, are requested [24–26].
